# Diagnosis *exjuvantibus* of a persistent pleural effusion

**DOI:** 10.3402/jchimp.v3i3-4.22466

**Published:** 2013-12-17

**Authors:** José M. Porcel

**Affiliations:** Pleural Diseases Unit, Department of Internal Medicine, Arnau de Vilanova University Hospital, Biomedical Research Institute of Lleida, Lleida, Spain

**Keywords:** uremic pleuritis, pleural effusion, dialysis

## Abstract

The diagnosis of uremia-associated effusion is one of exclusion. A patient with an unexplained chronic pleural exudate, which cleared with dialysis, is reported. The differential diagnosis of pleural effusions in patients with chronic kidney disease and the management of uremic pleuritis is briefly discussed.

A 48-year-old man was evaluated for a new pleural effusion. He had hypertension and a 2-year history of type 2 diabetes mellitus. Four months earlier, he had developed overt proteinuria, which was followed by a percutaneous kidney biopsy that demonstrated diabetic nephropathy. At that time, laboratory studies revealed a serum creatinine level of 1.5 mg/dL, blood urea nitrogen of 39 mg/dL, and an estimated glomerular filtration rate using the Modification of Diet in Renal Disease (MDRD) equation of 51 mL/min/1.73 m^2^. No pleural effusions were detected on a chest radiograph. Medications were metformin, insulin glargine, furosemide, and valsartan.

Most recently, the patient was hospitalized for a pulsatile mass on the right inner thigh with an associated ipsilateral lower extremity edema. On physical examination, the patient was afebrile, and decreased breath sounds and dullness to percussion on the right chest was noted. There was no elevation in jugular venous pressure, and cardiac examination was unremarkable. Chest radiograph revealed a right-sided pleural effusion occupying nearly half of the hemithorax (Fig. 1), although the patient had no respiratory complaints.

**Fig. 1:**
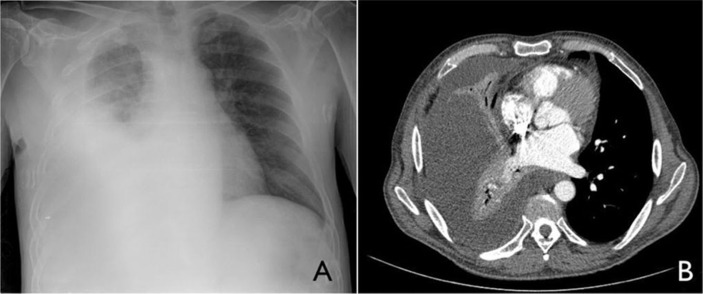
Initial chest radiograph (A) and CT (B) showing a large loculated right-sided pleural effusion.

Results of laboratory studies showed a serum creatinine level of 2.88 mg/dL, blood urea nitrogen of 58 mg/dL, an estimated glomerular filtration rate of 20 mL/min/1.73 m^2^, a leukocyte count of 8.7 × 10^9^/L, and a hemoglobin level of 8.5 mg/dL. Pleural fluid analysis showed: erythrocyte count 4,100/µL, leukocytes 1,064/µL (80% lymphocytes), glucose 104 mg/dL, protein 3.2 g/dL (serum 6 g/dL), lactate dehydrogenase 328 U/L (serum 324 U/L; upper normal limit for serum 480 U/L), cholesterol 66 mg/dL, albumin 1.2 g/dL (serum 2.3 g/dL), pH 7.43, adenosine deaminase 17.8 U/L, negative bacterial and mycobacterial cultures, and no malignant cells. An angio-CT with preventive interventions for contrast-induced nephropathy (i.e., isotonic intravenous fluids, N-acetylcysteine, and sodium bicarbonate) ruled out pulmonary embolism and displayed a large loculated right-sided pleural effusion. Kidney function tests did not change after CT imaging. Echocardiography showed left ventricular hypertrophy, and abdominal CT was not contributory. An ultrasonography of the legs detected a 6-cm superficial right femoral artery pseudoaneurism compressing the deep venous system.

Given the possibility of a non-specific pleuritis (e.g., viral pleuritis) and the contraindication for the use of non-steroidal anti-inflammatory drugs, an empirical tapering course of prednisone (initial dose of 30-mg daily) was instituted. The pleural effusion remained unchanged during follow-up visits at 6 weeks intervals. A thoracoscopic pleural biopsy was then planned, but soon after (4 months from the start of corticosteroid therapy), the patient developed signs of volume overload and deterioration of the renal function (blood urea nitrogen 55 mg/dL, creatinine 5.23 mg/dL, glomerular filtration rate 12.6 mL/min/1.73 m^2^). Consequently, corticosteroids were stopped and hemodialysis was promptly initiated. Later, the effusion gradually cleared and 4 months later virtually disappeared (Fig. 2), thus confirming its relationship with a uremic state. The *Kt*/*V*, a way of measuring hemodialysis adequacy, has always been above 1.4, as recommended by the current guidelines. The pseudoaneurism was subsequently repaired using endovascular techniques.

**Fig. 2:**
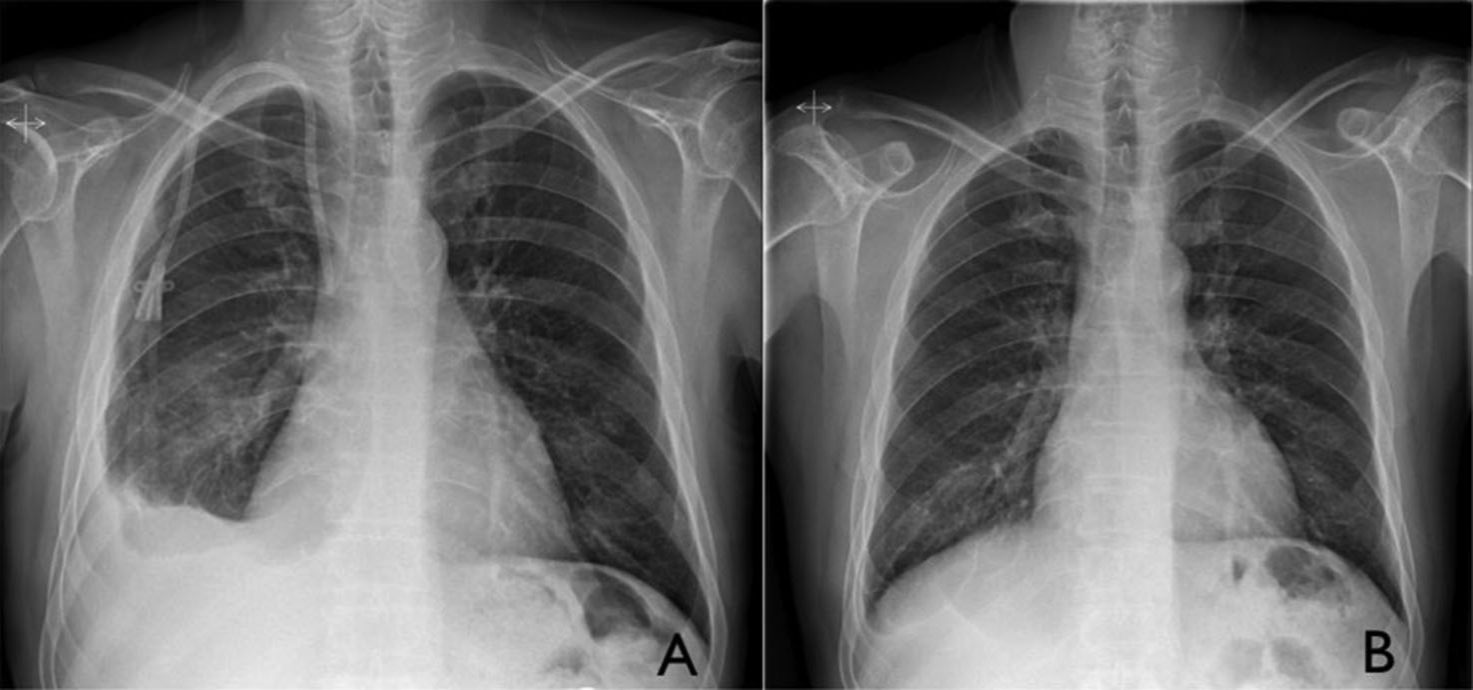
Gradual resolution of the pleural effusion, 1 month (A) and 4 months (B) after initiation of hemodialysis.

## Discussion

Patients with end-stage renal disease may develop pleural effusions due to many causes, including volume overload, heart failure, peritoneal dialysis (in all such cases the fluid is a transudate), infection, pulmonary embolism, malignancy, uremia, or following long-term hemodialysis.

Uremic pleuritis results from inflammation of the visceral and parietal pleural membranes and its pathogenesis is poorly understood (1). The incidence of fibrinous pleuritis, whether or not associated with pleural effusion, is reported to be 20–57% in patients who died of uremia (2). Half the patients are asymptomatic, while the remainder have fever, pleuritic chest pain, dyspnea, or non-productive cough (2). A close relationship between the degree of uremia and the occurrence of pleural effusions has not been found (3). Effusions are unilateral in 80% of patients and may be large. The pleural fluid is typically a serous or serosanguineous exudate with a predominance of either lymphocytes or neutrophils (4). Pleural biopsy, when performed, shows chronic fibrinous pleuritis. One third of patients have a concomitant pericardial effusion (4).

Uremic pleuritis, which is a diagnosis of exclusion, is strongly supported by the resolution of the effusion within several weeks or months of continued dialysis. However, in about 20% of cases the pleuritis persists, recurs or occasionally progresses to restrictive ventilatory dysfunction that needs decortication (1). A few anecdotal cases of refractory uremic pleuritis have improved with corticosteroids (5).
